# Metabolic Analyses and Evaluation of Antioxidant Activity in Purple Kohlrabi Sprouts after Exposed to UVB Radiation

**DOI:** 10.3390/antiox11081443

**Published:** 2022-07-25

**Authors:** Hyeon Ji Yeo, Soo-Yeon Lim, Chang Ha Park, Cha Young Kim, Ramaraj Sathasivam, Jae Kwang Kim, Sang Un Park

**Affiliations:** 1 Biological Resource Center, Korea Research Institute of Bioscience and Biotechnology (KRIBB), 181 Ipsin-gil, Jeongeup 56212, Korea; hjyeo@kribb.re.kr (H.J.Y.); kimcy@kribb.re.kr (C.Y.K.); 2 Department of Crop Science, Chungnam National University, 99 Daehak-ro, Yuseong-gu, Daejeon 34134, Korea; ramarajbiotech@gmail.com; 3 Department of Genetic Engineering and Graduate School of Biotechnology, College of Life Sciences, Kyung Hee University, Yongin 17104, Korea; sooyeon2324@khu.ac.kr; 4 Department of Biological Sciences, Keimyung University, 1095 Dalgubeol-daero, Dalseo-gu, Daegu 42601, Korea; pch3444@kmu.ac.kr; 5 Division of Life Sciences and Convergence Research Center for Insect Vectors, College of Life Sciences and Bioengineering, Incheon National University, Yeonsu-gu, Incheon 22012, Korea; 6 Department of Smart Agriculture Systems, Chungnam National University, 99 Daehak-ro, Yuseong-gu, Daejeon 34134, Korea

**Keywords:** kohlrabi sprouts, fatty acids, anthocyanins, hydrophilic metabolites

## Abstract

Various metabolites act as plant defense molecules due to their antioxidant abilities. This study aimed to investigate the influence of UVB irradiation on the accumulation of metabolites, including primary metabolites (sugar, sugar alcohols, amino acids, organic acids, and an amine) and secondary metabolites (anthocyanins, fatty acids, and phenolic acids), and its synergistic antioxidant ability, in purple kohlrabi sprouts. Metabolite analyses revealed a total of 92 metabolites in the sprouts. Specifically, the levels of most amino acids increased after 24 h of UVB treatment, and then slightly decreased in the kohlrabi sprouts. The levels of most sugars and sugar alcohols increased after 24 h of UVB treatment and then decreased. The levels of TCA cycle intermediates and phenolic acids gradually increased during the UVB treatment. Furthermore, the levels of some fatty acids gradually increased during the UVB treatment, and the levels of the other fatty acids increased after 6 h of UVB treatment and then decreased. In particular, the levels of most anthocyanins, known to be strong antioxidants, gradually increased after 24 h of UVB treatment. In the in vitro ABTS scavenging assay, UVB-treated purple kohlrabi sprouts showed increased scavenging ability. This may be attributed to the increased accumulation of metabolites acting as antioxidants, in response to UVB treatment. This study confirmed that UVB irradiation induced the alteration of primary and secondary metabolism in the kohlrabi sprouts.

## 1. Introduction

Being sessile organisms, plants are inevitably exposed to the ultraviolet (UV) radiation emitted from the sun. UVB (280–320 nm), which occupies a small part of the UV spectrum, can reach plants after crossing the ozone layer [1]. In response, plants have evolved light-mediated responses, such as alteration of morphology, physiology, and accumulation of metabolites involved in antioxidant defense [2]. In particular, plants can produce secondary metabolites, including flavonoids, fatty acids, and phenolic acids, reducing the oxidative stress and screening UV radiation [2,3]. 

In addition to UV, abiotic stresses, such as chilling, drought, dehydration, and salt, adversely affect crop productivity by limiting plant growth and altering metabolic activity. A mitigation strategy against these abiotic stresses has been passionately developed. For example, the application of nanoparticles has been found to enhance the chilling tolerance of sugarcane [4] as well as gamma irradiation enhanced the salinity tolerance in potato plantlets [5]. Elsheery and Cao reported partial shading could reduce the harmful effect of drought stress in mango (*Mangifera indica*) [6], and Elsheery et al. 2020 reported that nanoparticles, such as nano-zinc oxide and nano-silicon, improved the salinity tolerance in mango trees [7]. 

Primary metabolites, namely nucleic acids, proteins, fats, carbohydrates, and lipids, are commonly present in plant species and are necessary for their survival and development [8]. Furthermore, several amino acids can act as stress alleviating molecules, responding to a variety of stresses. In particular, proline is a proteinogenic amino acid involved in defense against salt, drought, and pathogen attack [9,10] and γ-aminobutyric acid (GABA) is a non-proteinogenic amino acid involved in defense against pathogen attack [9,10,11]. Moreover, primary metabolites are used as precursors or intermediates for secondary metabolites and supply energy for secondary metabolism [12]. 

Plant secondary metabolites play a significant role in defense mechanisms triggered by various abiotic and biotic stresses [13]. Phenolic compounds, including flavonoids (e.g., anthocyanin) and phenylpropanoids, have been reported to be involved in defense against UV radiation exposure, due to their ability to absorb UVB radiation and thus limit its penetration into leaf epidermal cells [14]. In particular, anthocyanins can be triggered or upregulated in the plants responding to UV irradiation, and function as UV absorbents and antioxidants [15]. Anthocyanin phenylacylation, with sinapoyl, feruloyl cinnamoyl, caffeoyl, and 4-coumaroyl moieties, improves their biological functions as UVB protectants [16], since acylation confers increased chemical stability [17] and acylated anthocyanins strongly absorb UV radiation [15,16]. 

Furthermore, fatty acids can contribute to plant defense as biosynthetic precursors for jasmonate, which regulates plant defense or cuticular constituents important for plant basal immunity. Fatty acids and their derivatives can be critical for triggering systemic acquired resistance and *R* gene-mediated resistance in plants, and their breakdown molecules (oxylipins) are either directly antimicrobial or indirectly participate in resistance against pathogens by mediating defense responses [18]. 

Kohlrabi (*Brassica oleracea* var. *gongylodes*) is a *Brassica* vegetable characterized by a bulb-shaped stem, and is commercially consumed due to its health-enhancing properties originating from its diverse metabolites [12]. A previous study has mainly focused on the UVB-induced secondary metabolite accumulation in kohlrabi sprouts [19]. To our knowledge, however, there have been no studies on the effect of UVB radiation on overall metabolic changes (both in primary and secondary metabolites) in purple kohlrabi sprouts. The objectives of the current study were to investigate the alteration in primary and secondary metabolite composition, including anthocyanins, fatty acids, amino acids, carbohydrates, sugar alcohols, phenolic acids, and organic acids, in UVB-treated purple kohlrabi sprouts via metabolite analyses (liquid chromatography–mass spectrometry (LC-MS), gas chromatography–time–of–flight mass spectrometry (GC-TOFMS), and gas chromatography–mass spectrometry GC-MS).

## 2. Materials and Methods

### 2.1. Plant Materials

To establish sprouts, 100 seeds were soaked with 70% ethanol (*v*/*v*) for 2 min and then washed with distilled water (DW), followed by immersion in DW for 1 day. Afterwards, the 100 seeds were planted into a plastic pot containing vermiculite. A total of 27 such pots, each containing 100 seeds, were placed in a growth chamber equipped with fluorescent lamps with a flux rate of 35 μmol·m^−2^·s^−1^ at 25 °C for 100 days. One pot containing 100 sprouts was regarded as one biological replicate, and further studies were performed with three biological replicates. Afterwards, sprouts on 3 pots were harvested as control at 0 h. A total of 12 pots were placed in a growth chamber equipped with two Philips PL-L 36W/01/4P UVB lamps (305−315 nm (λpeak = 311 nm); Philips, The Netherlands)) at 25 °C, and the other 12 pots were still located in the growth chamber with fluorescent lamps. At time points of 6 h, 12 h, 24 h, and 48 h, three pots each from the growth chamber with UVB lamps and the growth chamber with fluorescent lamps were harvested.

### 2.2. Extraction of Kohlrabies for Anthocyanin Quantification and Antioxidant Activity

The kohlrabi sprouts were freeze-dried and grounded. From each sample, 50 mg of the powder was extracted with 5% formic acid (1 mL) and vortexed for 5 min. The sample was then sonicated for 30 min and centrifuged at 8000 rpm and 4 °C for 15 min. 

### 2.3. HPLC and LC-MS Analyses

HPLC analyses were carried out using an HPLC system with a Jasco intelligent model PU-2080 pump (Jasco, Tokyo, Japan), a Waters 2080 pump (Waters, Milford, MA, USA), and a UV-2075 UV detector (Jasco, Tokyo, Japan). An Agilent SB-C18 (4.6 × 150 mm, 5 µm) column was used. The flow rate was 1 mL/min, and the injection volume was 10 µL. Solvent A consisted of 5% formic acid in water, and solvent B was acetonitrile. The gradient elution system was as follows: 0–1 min: 5% B; 1–20 min 5–15% B; 20–28 min:15% B; 28–33 min: 15–18% B; 33–38 min: 18% B; 38–40 min: 18–19% B; 40–42 min: 19–20% B; 42–44 min: 20–21% B; 44–49 min: 21% B; 49–50 min: 21–5% B; 50–70 min: 5% B. The phases were allowed to equilibrate between injections. Anthocyanin peaks were detected at 520 nm. The conditions for MS analysis were the same as those described above, using the QTRAP 4500 Ultra High Performance Hybrid LC-MS/MS system. Spectra range was m/z 5 to 2000, and the source voltage was −4.5 kV to 5.5 kV

### 2.4. Determination of Total Phenolic Content (TPC)

The total phenolic content of purple kohlrabi sprouts grown without UVB irradiation for 0 h, 6 h, 12 h, 24 h, and 48 h and purple kohlrabi sprouts irradiated with UVB radiation for 6 h, 12 h, 24 h, and 48 h was measured using the Folin–Ciocalteu method [20]. In brief, 250 µL of extract was added to a tube containing 750 µL of Folin–Ciocalteu reagent and 3.4 mL of DW, and vortexed for 10 s, followed by incubation at 25 °C for 5 min. Afterwards, 200 µL of 7.5% sodium carbonate solution (*w*/*v*) was added to the tube, followed by incubation in darkness at 25 °C for 60 min. The absorbance of the resulting mixtures was measured at 765 nm against a blank (MeOH). The total phenolic content was calculated and depicted as gallic acid equivalents (GAE).

### 2.5. ABTS Assay

ABTS solution (8.5 mM) and potassium persulfate solution (3.5 mM) were mixed in equal quantities and kept in darkness at 25 °C for 24 h for the assay. After dilution with DW, 200 µL of ABTS solution was added to 50 µL of sample extracts, followed by measurement of absorbance at 750 nm. MeOH (50%) (*v*/*v*) was used as a negative control, and Trolox was used as a positive control. ABTS radical scavenging activity was calculated using the following equation: ABTS scavenging activity (%) = (1 − sample absorbance/control absorbance) × 100.

### 2.6. Lipid Extraction and Chemical Derivation

From each sample (10 mg), lipid metabolites were extracted using hexane/diethyl ether (9:1) and vortexed at 2500 rpm for 10 min. After centrifugation at 1500× *g* for 5 min, the sample extract was transferred to a new tube, and the pellet was re-extracted two more times using the same method. The lipid extracts were derivatized to methyl esters before further analysis. In brief, lipid extracts were derivatized in 1N KOH in EtOH (4 mL), and 14% BF3 MeOH (5 mL) was added for 5 min at 80 °C. Subsequently, 3 mL of saturated NaCl was added, and the lipids were extracted by mixing with 1 mL of hexane. Then, the upper layer was mixed with 100 µL of internal standard (0.05 mg/mL of eicosane) and hexane (200 µL) for GC spectroscopy.

### 2.7. Gas Chromatography–Mass Spectrometry (GC-MS) Analysis 

Gas chromatography was performed with an Agilent 6890N GC main frame (Agilent, Santa Clara, CA, USA) and an Agilent 7820A GC equipped with 5977E MSD for GC-MS. Each chromatography experiment used DB-5MS (60 m × 0.32 mm I.D * 0.25 um film thickness, Agilent, USA). For samples that were only trans-methylated, 1 µL aliquots were injected into the GC via splitless injection, and the GC oven temperature program was set as follows: start at 50 °C, increase at a rate of 4 °C/min to 190 °C, decrease at a rate of 0.5 °C/min to 180 °C, hold for 5 min, increase at a rate of 4 °C/min to 280 °C, increase at a rate of 10 °C/min to 300 °C, and hold at this temperature for 5 min. Ionization energy of 70 eV was used for GC-MS. After performing the total ion chromatography (TIC) of each sample, identification was conducted by comparing retention time using the Chemstation software library.

### 2.8. GC-TOFMS Analysis

Dried powder (10 mg) of purple kohlrabi sprouts grown without UVB irradiation for 0 h, 6 h, 12 h, 24 h, and 48 h and purple kohlrabi sprouts irradiated with UVB radiation for 6 h, 12 h, 24 h, and 48 h were mixed with 0.45 mL of methanol with ribitol (0.2 g L^−1^) at 1300 rpm and 37 °C for 1 min and centrifuged at 12,000 rpm for 20 min. The supernatant was transferred to a fresh tube containing 190 μL of chloroform and 480 μL of DW, and was gently vortexed for 15 s, followed by centrifugation at 12,000 rpm for 10 min. Polar phase (450 μL) was collected and dried using a concentrator. Thereafter, 40 μL of methoxyaminhydrochlorid-pyridine (30 g L^−1^) was added and shaken at 1000 rpm and 37 °C for 2 h, followed by the addition of 70 μL of N-Methyl-N-(trimethylsilyl)trifluoroacetamide. The mixtures were then shaken at 1000 rpm and 37 °C for 0.5 h, and transferred to a vial for further analysis. The equipment and conditions for the analysis were based on the protocol established in our previous study [21]. Metabolite identification was carried out with selected ions and Chroma-TOF software [21].

### 2.9. Statistical Analysis

The anthocyanin and fatty acid data were analyzed using SAS software version 9.4 following a *t*-test. In order to analyze the quantitative data collected in this study, MetaboAnalyst 5.0 (http://www.metaboanalyst.ca/ (accessed on 1 May 2022)) was used for the principal component analysis (PCA), partial least-squares discriminant analysis (PLS-DA), heat map, and correlation analysis with auto-scaling.

## 3. Results

### 3.1. Anthocyanin Analysis

A total of 18 cyanidins were identified in UVB-treated purple kohlrabi sprouts (Table 1 and Figure S1). In particular, the levels of cyanidin 3-(caffeoyl)diglucoside-5-glucoside, cyanidin 3-(sinapoyl)diglucoside-5-glucoside, cyanidin 3-(feruloyl)(feruloyl)diglucoside-5-glucoside, cyanidin 3-(sinapoyl)(p-coumaroyl)diglucoside-5-glucoside, cyanidin 3,5-diglucoside, cyanidin 3-diglucoside-5-glucoside, cyanidin 3-(sinapoyl)diglucoside-5-glucoside, and cyanidin 3-(sinapoylloyl)(feruloyl)diglucoside-5-glucoside increased after 24 h UVB treatment, and cyanidin 3-diglucoside, cyanidin 3-(caffeoyl)(p-coumaroyl)diglucoside-5-glucoside, cyanidin 3-(glycopyranosyl-sinapoyl)diglucoside-5-glucoside, and cyanidin 3-(sinapoyl)diglucoside-5-glucoside started to accumulate after 24 h UVB treatment, compared to sprouts grown without UVB treatment for 24 h. Furthermore, cyanidin 3-(p-hydroxybenzoyl)(oxaloyl)diglucoside-5-glucoside began to be produced after 48 h UVB treatment.

### 3.2. Fatty Acid Analysis

A total of 23 fatty acids were identified in UVB-treated purple kohlrabi sprouts (Table 2 and Figure S2). After 24 h UVB treatment, the levels of 2,6,11-trimethyldodecane, 2,6,10-trimethyltetradecane, ethyl hexadecanoate, 9,12-octadecadienoic acid ethyl ester, linolenic acid, cis-13-eicosenoic acid, and 2-methyl eicosane increased, compared to sprouts grown for 24 h without UVB treatment, and similarly, the levels of farnesene and 2,3,5,8-tetramethyldecane, increased in sprouts grown for 48 h under UVB treatment. In contrast, the levels of 1-propylundecylmethoxyacetate, methyl linolate, methyl 13-eicosenoate, and methyl nervonate, decreased during UVB treatment, or were lower than those of spouts grown without UVB treatment.

### 3.3. Metabolite-Specific Profiling of UVB-Treated Purple Kohlrabi Sprouts

**Figure 1:**
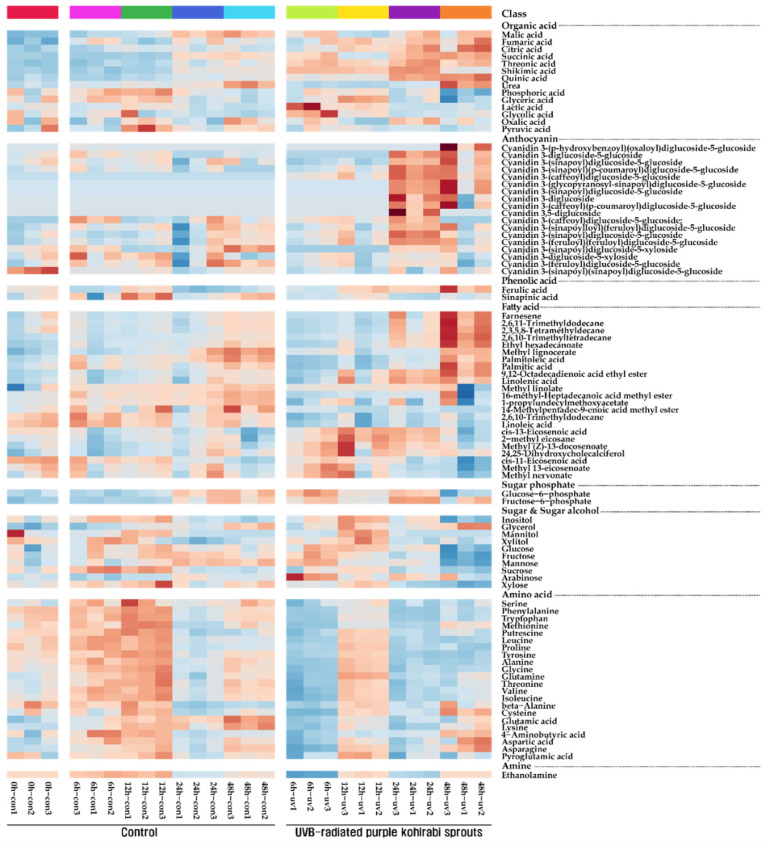
Heatmap representing changes in metabolite composition of purple kohlrabi sprouts in response to UVB irradiation. The colors indicate the relative content of each metabolite identified in control, purple kohlrabi sprouts grown without UVB treatment for 0 h, 6 h, 12 h, 24 h, and 48 h, and test, purple kohlrabi sprouts irradiated with UVB radiation for 6 h, 12 h, 24 h, and 48 h.

One amine, two phenolic acids, two sugar phosphates, four sugar alcohols, six carbohydrates, 14 organic acids, and 22 amino acids were identified in purple UVB-treated purple kohlrabi sprouts using GC-TOFMS (Figure 1 and Figure S3). The levels of amino acids (serine, phenylalanine, tryptophan, methionine, putrescine, leucine, proline, tyrosine, alanine, glycine, glutamine, threonine, valine, isoleucine, beta-alanine, cysteine, glutamic acid, lysine, 4-aminobutyric acid, aspartic acid, pyroglutamic acid, and asparagine) gradually increased and then slightly decreased in the sprouts grown without UVB treatment. However, the levels of these amino acids increased after 12 h UVB treatment and then decreased after 24 h UVB treatment. Furthermore, the levels of the majority of carbohydrates and sugar alcohols increased after 24 h UVB treatment and then decreased. The levels of TCA cycle intermediates (malic acid, fumaric acid, citric acid, and succinic acid) and phenolic acid (ferulic acid) gradually increased during the UVB treatment.

**Figure 2:**
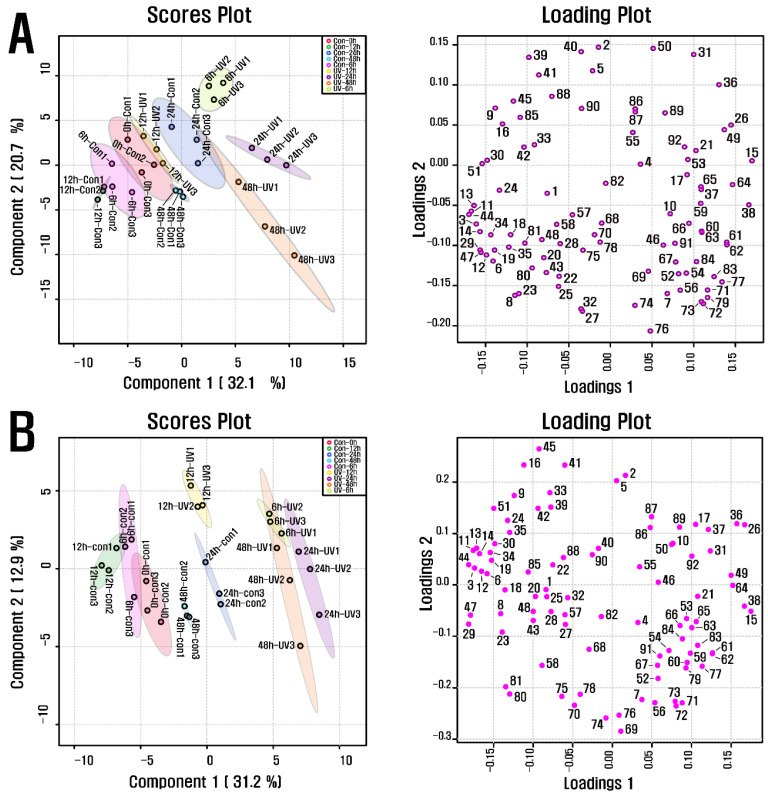
(**A**) Scores and loading plots of PCA model for metabolites present in UVB-irradiated purple kohlrabi sprouts and (**B**) Scores and loading plots of PLS-DA model for metabolites present in UVB-irradiated purple kohlrabi sprouts. Control, purple kohlrabi sprouts grown without UVB treatment for 0 h, 6 h, 12 h, 24 h, and 48 h; test, purple kohlrabi sprouts irradiated with UVB radiation for 6 h, 12 h, 24 h, and 48 h. Number: 1, Pyruvic acid; 2, Lactic acid; 3, Alanine; 4, Oxalic acid; 5, Glycolic acid; 6, Valine; 7, Urea; 8, Ethanolamine; 9, Phosphoric acid; 10, Glycerol; 11, Leucine; 12, Isoleucine; 13, Proline; 14, Glycine; 15, Succinic acid; 16, Glyceric acid; 17, Fumaric acid; 18, Serine; 19, Threonine; 20, β-Alanine; 21, Malic acid; 22, Aspartic acid; 23, Methionine; 24, Pyroglutamic acid; 25, 4-Aminobutyric acid; 26, Threonic acid; 27, Cysteine; 28, Glutamic acid; 29, Phenylalanine; 30, Xylose; 31, Arabinose; 32, Asparagine; 33, Xylitol; 34, Putrescine; 35, Glutamine; 36, Shikimic acid; 37, Citric acid; 38, Quinic acid; 39, Fructose; 40, Mannose; 41, Glucose; 42, Mannitol; 43, Lysine; 44, Tyrosine; 45, Inositol; 46, Ferulic acid; 47, Tryptophan; 48, Sinapinic acid; 49, Fructose-6-phosphate; 50, Glucose-6-phosphate; 51, Sucrose; 55, Cyanidin 3-(caffeoyl)diglucoside-5-glucoside; 57, Cyanidin 3-(feruloyl)diglucoside-5-glucoside; 58, Cyanidin 3-diglucoside-5-xyloside; 68, Cyanidin 3-(sinapoyl)(sinapoyl)diglucoside-5-glucoside; 69, Cyanidin 3-(sinapoyl)diglucoside-5-xyloside; 70, 2,6,10-Trimethyldodecane; 71, Farnesene; 72, 2,6,11-Trimethyldodecane; 73, 2,3,5,8-Tetramethyldecane; 74, Palmitoleic acid; 75, 1-propylundecylmethoxyacetate; 76, Palmitic acid; 77, 2,6,10-Trimethyltetradecane; 78, 14-Methylpentadec-9-enoic acid methyl ester; 79, Ethyl hexadecanoate; 80, Linoleic acid; 81, Methyl linolate; 82, 16-methyl-Heptadecanoic acid, methyl ester; 83, 9,12-Octadecadienoic acid, ethyl ester; 84, Linolenic acid; 85, cis-11-Eicosenoic acid; 86, cis-13-Eicosenoic acid; 87, 2-methyl eicosane; 88, Methyl 13-eicosenoate; 89, Methyl (Z)-13-docosenoate; 90, Methyl nervonate; 91, Methyl lignocerate; 92, 24,25-Dihydroxycholecalciferol.

PCA was carried out to investigate the metabolite alteration in purple kohlrabi sprouts in response to UVB treatment of various durations (Figure 2). The results showed a separation between the groups UVB-6 h and UVB-24 h, representing the purple kohlrabi sprouts treated with UVB radiation and the duration, and groups Con-0 h, Con-6 h, Con-12 h, Con-24 h, and Con-48 h, representing the purple kohlrabi sprouts not treated with UVB radiation and the duration. This separation was attributed to changes in the levels of cyanidins, fatty acids, organic acids, and amino acids. Subsequently, according to the PLS-DA, a clear separation between the groups UV-6 h, UV-12 h, UV-24 h, and UV-48 h and groups Con-0 h, Con-6 h, Con-12 h, Con-24 h, and Con-48 h was observed, and it was because of changes in levels of organic acids (threonic acid, shikimic acid, succinic acid, quinic acid, and fumaric acid), amino acids (phenylalanine, tryptophan, tyrosine, alanine, leucine, isoleucine, and glycine), fatty acids (methyl linolate and linoleic acid), and cyanidin 3-(caffeoyl)diglucoside-5-glucoside, with VIP score > 1.4, which was consistent with PCA results. 

**Figure 3:**
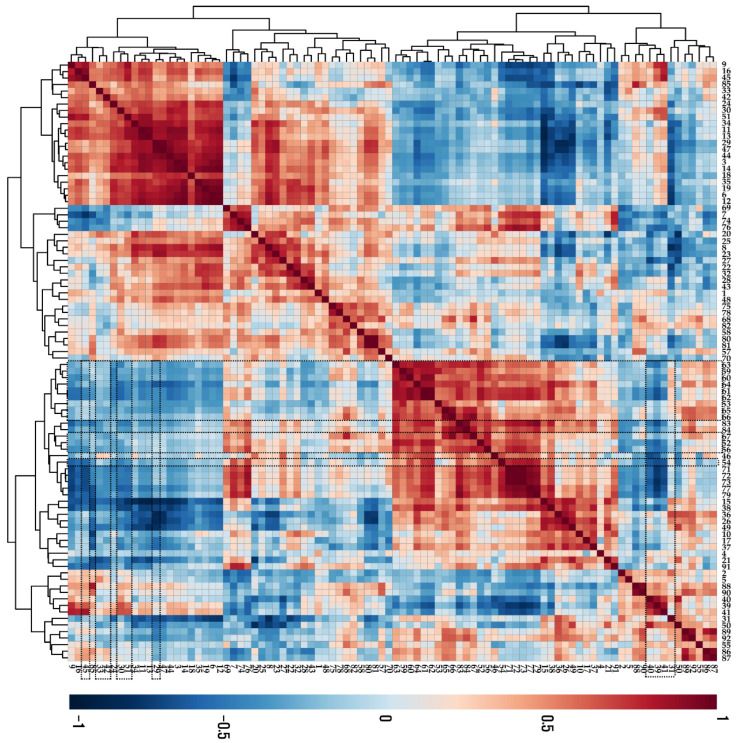
Correlation matrix of metabolites present in UVB-irradiated purple kohlrabi sprouts. Control, purple kohlrabi sprouts grown without UVB treatment for 0 h, 6 h, 12 h, 24 h, and 48 h; test, purple kohlrabi sprouts irradiated with UVB radiation for 6 h, 12 h, 24 h, and 48 h. Number: 1, Pyruvic acid; 2, Lactic acid; 3, Alanine; 4, Oxalic acid; 5, Glycolic acid; 6, Valine; 7, Urea; 8, Ethanolamine; 9, Phosphoric acid; 10, Glycerol; 11, Leucine; 12, Isoleucine; 13, Proline; 14, Glycine; 15, Succinic acid; 16, Glyceric acid; 17, Fumaric acid; 18, Serine; 19, Threonine; 20, β-Alanine; 21, Malic acid; 22, Aspartic acid; 23, Methionine; 24, Pyroglutamic acid; 25, 4-Aminobutyric acid; 26, Threonic acid; 27, Cysteine; 28, Glutamic acid; 29, Phenylalanine; 30, Xylose; 31, Arabinose; 32, Asparagine; 33, Xylitol; 34, Putrescine; 35, Glutamine; 36, Shikimic acid; 37, Citric acid; 38, Quinic acid; 39, Fructose; 40, Mannose; 41, Glucose; 42, Mannitol; 43, Lysine; 44, Tyrosine; 45, Inositol; 46, Ferulic acid; 47, Tryptophan; 48, Sinapinic acid; 49, Fructose-6-phosphate; 50, Glucose-6-phosphate; 51, Sucrose; 55, Cyanidin 3-(caffeoyl)diglucoside-5-glucoside; 57, Cyanidin 3-(feruloyl)diglucoside-5-glucoside; 58, Cyanidin 3-diglucoside-5-xyloside; 68, Cyanidin 3-(sinapoyl)(sinapoyl)diglucoside-5-glucoside; 69, Cyanidin 3-(sinapoyl)diglucoside-5-xyloside; 70, 2,6,10-Trimethyldodecane; 71, Farnesene; 72, 2,6,11-Trimethyldodecane; 73, 2,3,5,8-Tetramethyldecane; 74, Palmitoleic acid; 75, 1-propylundecylmethoxyacetate; 76, Palmitic acid; 77, 2,6,10-Trimethyltetradecane; 78, 14-Methylpentadec-9-enoic acid methyl ester; 79, Ethyl hexadecanoate; 80, Linoleic acid; 81, Methyl linolate; 82, 16-methyl-Heptadecanoic acid, methyl ester; 83, 9,12-Octadecadienoic acid, ethyl ester; 84, Linolenic acid; 85, cis-11-Eicosenoic acid; 86, cis-13-Eicosenoic acid; 87, 2-methyl eicosane; 88, Methyl 13-eicosenoate; 89, Methyl (Z)-13-docosenoate; 90, Methyl nervonate; 91, Methyl lignocerate; 92, 24,25-Dihydroxycholecalciferol.

According to the results from the Pearson correlation analysis (Figure 3), most anthocyanins were negatively correlated with phenylalanine, which is an initial precursor for phenylpropanoids and flavonoids. Furthermore, these anthocyanins were negatively correlated with sugars (sucrose, arabinose, glucose, fructose, mannose, and xylose) and sugar alcohols (xylitol, mannitol, and inositol), which can be used as energy sources for anthocyanin biosynthesis. 

### 3.4. Total Phenolic Content and Antioxidant Assay

The total phenolic content in the purple kohlrabi sprouts varied according to the duration of UVB treatment, and ranged from 0.60 to 0.67 mg/g dry weight (Table 3). The phenolic compound accumulation showed a gradual increase with increasing duration of UVB treatment, and the highest level was observed after 12 h of UVB treatment (Table 3). The ABTS free radical scavenging activity was measured using methanol extracts from purple kohlrabi sprouts (Table 3). The scavenging activities in purple kohlrabi sprouts grown without UVB treatment were not significantly different compared with control. In contrast, the activity gradually increased during the UVB treatment. This might be due to the increased accumulation of secondary metabolites in response to UVB treatment, since ABTS activity was positively correlated with most acylated anthocyanins and fatty acids (Figure S4). 

## 4. Discussion

In this study, the effect of UVB radiation on the metabolite composition of purple kohlrabi sprouts was investigated using LC-MS, HPLC, GC-MS, and GC-TOFMS. A total of 92 metabolites, including 1 amine, 2 phenolic acids, 2 sugar phosphates, 4 sugar alcohols, 6 carbohydrates, 14 organic acids, 22 amino acids, 18 cyanidins, and 23 fatty acids, were identified in kohlrabi sprouts exposed to UVB radiation for 0, 6, 12, 24, and 48 h. According to secondary metabolite analysis, the levels of anthocyanins and fatty acids increased after UVB irradiation. Previously, UVB irradiation has been reported to induce phenylpropanoid and flavonoid biosynthesis in plant species [15]. These phenolics, including phenolic acids and anthocyanins, are mainly distributed in the plant epidermis and can function as antioxidants as well as UVB absorbers [22,23]. UVB irradiation resulted in an increase in the levels of phenolic compounds in many *Brassica* vegetables. For example, the levels of flavonol glycosides and hydroxycinnamic acid derivatives in the leaves of *B*. *oleracea* var. *sabellica* [24], flavonol glycosides in the leaves of *B*. *napus* [25], anthocyanins in *B*. *rapa* [26], and *B*. *oleracea* [27], and flavonoids in the leaves of *Sinapis alba* and *Nasturtium officinale* [28], increased in response to UVB treatment. Furthermore, phenylacylated anthocyanins were mainly identified in the sprouts, and their levels dramatically increased during UVB treatment in this study. Previously, the UVB protectant function of anthocyanins has been reported to be enhanced via phenyaclylation, which increases their stability and absorption of UVB radiation [15,16,17]. In addition, biotic stresses increased the production of secondary metabolites in plants. For example, Potato virus X (PVX) infection induced the production of phenolic compounds, such as phenolic acids and flavonoids in tobacco [29] as well as wheat against *Rhopalosiphum Padi* produced phenolic compounds [30]. Therefore, this study suggests that UVB irradiation upregulates anthocyanin biosynthesis and anthocyanin phenylacylation in purple kohlrabi sprouts.

Plant species can maintain metabolism and homeostasis via the modulation of fluidity and stability of plasma membrane under unfavorable situations [31], and saturated/unsaturated fatty acids play a particularly crucial role in membrane rigidity [32]. In this study, the levels of fatty acids increased in purple kohlrabi sprouts upon exposure to UVB radiation. This finding implies that the increase in the levels of fatty acids may help sustain the metabolism and homeostasis in purple kohlrabi sprouts exposed to UVB radiation. Similarly, Zhou et al. [32] have suggested that UVB radiation induces an increase in the levels of fatty acids, organic acids, and flavonoids in the leaves of *Rhododendron chrysanthum* irradiated by UVB radiation [31], and Moorthy and Kathiresan [33] have reported an increase in the levels of myristoleic acid in mangroves under high dose of UVB radiation [33].

In this study, the levels of phenylalanine, which is an initial precursor for flavonoids (anthocyanins), gradually decreased during UVB exposure, but the levels of anthocyanins increased in a time-dependent manner for the duration of the UVB treatment. This negative correlation may reflect a metabolic precursor demand to support anthocyanin biosynthesis. Similarly, rhizobacteria inoculation induced a greater accumulation of secondary metabolites, including phenolic acids and flavonoids, while the levels of phenylalnine were depleted in *B*. *oleracea* sprouts [34]. Additionally, Yeo et al. [35], reported that the depleted endogenous pools of phenylalanine in *Scutellaria baicalensis* plantlets exposed to light-emitting diodes (LEDs) showed an increased accumulation of flavones [35], and Park et al., [12] reported a negative correlation between the levels of phenylalanine and anthocyanins in purple kohlrabi [12]. Furthermore, anthocyanins were negatively correlated with sugars (sucrose, arabinose, glucose, fructose, mannose, and xylose) in the UVB-exposed purple kohlrabi sprouts, indicating energy demand for anthocyanin biosynthesis. This finding was consistent with the previous studies reporting that the more rapidly depleted levels of sugars in cell cultures of *Papaver somniferum* treated with a fungal elicitor indicated an increased accumulation of alkaloids [36], as well as the depleted levels of sucrose, xylose, and glucose in hairy root cultures of *S. baicalensis* overexpressing ZmLc, showed an increase in flavones [37]. The sugars were negatively correlated with anthocyanins in purple kohlrabi [12]. 

UVB radiation has been reported to induce the production of harmful molecules, such as reactive oxygen species (ROS), thus damaging plant organs and primary metabolites, and reducing photosynthesis, all of which are necessary for plant life [38,39]. In response to UVB stress, plants can activate secondary metabolism, leading to increased levels of secondary metabolites that act as antioxidants by scavenging ROS [40]. In this study, ABTS analysis showed increased scavenging activities in UVB-treated purple kohlrabi sprouts, compared to purple kohlrabi sprouts grown without UVB treatment. This might be due to the increased accumulation of secondary metabolites in response to UVB treatment, since ABTS activity was positively correlated with the levels of most acylated anthocyanins. These findings are in agreement with previous studies showing synergistic antioxidant activities originating from secondary metabolites in Korean mint [41] and downy lavender [42].

## 5. Conclusions

This study aimed to investigate the effect of UVB radiation on changes in the composition of primary and secondary metabolites in purple kohlrabi sprouts. Notably, UVB irradiation induced an overall alteration in metabolite accumulation in the sprouts. According to the primary metabolite analysis, the levels of most amino acids and sugars, used as energy sources or metabolic precursors for secondary metabolites, decreased, but the levels of proline and GABA, which play a crucial role in plant defense, increased during UVB irradiation in purple kohlrabi sprouts. Furthermore, an increase in the levels of secondary metabolites, such as anthocyanins, phenolic acids, and fatty acids, that function as antioxidants, UV absorbers, or structural components of membrane, was observed. ABTS free radical scavenging ability was also found to be increased during UVB irradiation. The increased scavenging capability may be due to an increase in the levels of secondary metabolites in UVB-treated purple kohlrabi sprouts. Thus, this study indicates that UVB irradiation can induce an overall alteration in metabolite composition, and particularly lead to an increase in the levels of proline, GABA, anthocyanins, fatty acids, and organic acids, which are involved in plant defense, in purple kohlrabi sprouts. 

## Figures and Tables

**Table 1:** Quantitative analysis of anthocyanin in UVB-treated purple kohlrabi sprouts.

Anthocyanin (μg/mL)	Rt ^1^ (min)	[M]^+^ (m/z)	MS/MS		Non-UV Treated				UV Treated			
				0 h	6 h	12 h	24 h	48 h	6 h	12 h	24 h	48 h
Cyanidin 3-diglucoside-5-glucoside	8.82	773	611/449/287	0.56 ± 0.23	0.90 ± 0.29	0.72 ± 0.17	ND ^2^	0.42 ± 0.13 ***^3^	ND	ND	1.68 ± 0.31 **	1.28 ± 0.85
Cyanidin 3,5-diglucoside	9.51	611	449/287	ND	ND	ND	ND	ND	ND	ND	0.52 ± 0.31	ND
Cyanidin 3-(p-hydroxybenzoyl)(oxaloyl)diglucoside-5-glucoside	10.91	965	803/449/287	ND	0.39 ± 0.24	ND	ND	ND	ND	ND	ND	7.34 ± 5.41
Cyanidin 3-(caffeoyl)diglucoside-5-glucoside	12.39	935	449/287	ND	3.87 ± 0.70 **	0.54 ± 0.37	2.23 ± 2.20	2.07 ± 1.00	2.27 ± 0.56 **	2.09 ± 0.90 *	3.88 ± 0.71 **	ND
Cyanidin 3-(sinapoyl)diglucoside-5-glucoside	15.94	979	817/449/287	3.11 ± 0.89	2.41 ± 0.49 **	2.96 ± 0.64 **	2.26 ± 2.27	2.76 ± 1.49	1.20 ± 0.44	1.58 ± 0.71	4.45 ± 0.16 ***	4.78 ± 2.20 *
Cyanidin 3-(feruloyl)diglucoside-5-glucoside	18.14	949	787/449/287	2.93 ± 0.94	4.02 ± 1.70 *	3.95 ± 0.59 ***	2.40 ± 2.21	1.81 ± 0.68 *	2.71 ± 0.62 **	2.38 ± 1.10 *	2.80 ± 0.18 ***	2.24 ± 1.05
Cyanidin 3-diglucoside-5-xyloside	19.96	743	611/419/287	0.80 ± 0.40	1.28 ± 0.57	1.40 ± 0.41 *	0.63 ± 0.93	2.16 ± 1.00	0.43 ± 0.09	ND	0.42 ± 0.22	ND
Cyanidin 3-diglucoside	21.24	611	287	ND	ND	ND	ND	ND	ND	ND	0.47 ± 0.26	0.13 ± 0.39
Cyanidin 3-(caffeoyl)(p-coumaroyl)diglucoside-5-glucoside	23.24	1081	919/449	ND	ND	ND	ND	ND	ND	ND	0.33 ± 0.11	0.24 ± 0.47
Cyanidin 3-(glycopyranosyl-sinapoyl)diglucoside-5-glucoside	25.04	1141	979/817/449	ND	ND	ND	ND	ND	ND	ND	1.29 ± 0.12 **	1.24 ± 1.09
Cyanidin 3-(sinapoyl)diglucoside-5-glucoside	26.76	1141	817/449/287	ND	ND	ND	ND	ND	ND	ND	1.00 ± 0.12 *	1.03 ± 0.86
Cyanidin 3-(sinapoyl)(p-coumaroyl)diglucoside-5-glucoside	27.74	979	963/449	1.19 ± 0.60	1.62 ± 0.97	1.48 ± 0.37 *	0.95 ± 1.20	ND	0.79 ± 0.32	1.67 ± 0.96	4.77 ± 0.71 ***	3.21 ± 1.83
Cyanidin 3-(caffeoyl)diglucoside-5-glucoside	28.86	979	773/449/287	ND	ND	ND	ND	ND	0.77 ± 0.18	1.00 ± 0.66	2.45 ± 0.37 **	1.63 ± 0.85
Cyanidin 3-(sinapoyl)diglucoside-5-glucoside	34.65	979	817/655/449/287	14.66 ± 6.39	11.43 ± 1.73 ***	13.61 ± 4.00 **	12.35 ± 10.52	21.31 ± 2.66 ***	17.18 ± 3.10 ***	19.44 ± 6.70 **	34.99 ± 2.14 ***	17.05 ± 5.29 **
Cyanidin 3-(feruloyl)(feruloyl)diglucoside-5-glucoside	39.05	1125	963/449	6.93 ± 1.77	8.00 ± 1.34 ***	8.46 ± 1.29 ***	6.87 ± 5.40	10.24 ± 0.19 ***	8.20 ± 1.47 ***	10.08 ± 3.31 **	14.83 ± 0.27 ***	9.75 ± 3.99 *
Cyanidin 3-(sinapoylloyl)(feruloyl)diglucoside-5-glucoside	39.90	1125	993/899/449	9.83 ± 2.25	12.60 ± 2.00 ***	13.43 ± 2.08 ***	10.56 ± 7.88	15.51 ± 1.22 ***	11.22 ± 2.06 ***	11.55 ± 3.74 **	17.86 ± 0.60 ***	14.88 ± 6.75 *
Cyanidin 3-(sinapoyl)(sinapoyl)diglucoside-5-glucoside	40.55	1185	1023/449	41.40 ± 10.92	52.13 ± 9.40 ***	54.48 ± 8.43 ***	45.40 ± 32.25	61.37 ± 7.29 ***	45.68 ± 6.83 ***	43.29 ± 14.12 **	51.53 ± 1.15 ***	36.66 ± 15.07 *
Cyanidin 3-(sinapoyl)diglucoside-5-xyloside	42.54	949	817/419/287	2.57 ± 0.66	3.21 ± 0.63 **	0.45 ± 0.18	1.77 ± 1.61	5.06 ± 0.91 ***	ND	ND	3.19 ± 0.06 ***	2.82 ± 1.22 *

^1^ Rt, Retention time. ^2^ ND, Not detected. ^3^ The t-test showed statistically significant difference (*** *p* <0.001, ** *p* <0.01, * *p* <0.05) compared with control (0 h).

**Table 2:** Compositions (%) of fatty acids in UVB-treated purple kohlrabi sprouts.

Fatty Acid (%)	Molecular Formula	Rt (min)		Non-UV Treated				UV Treated			
			0 h	6 h	12 h	24 h	48 h	6 h	12 h	24 h	48 h
2,6,10-Trimethyldodecane	C_15_H_32_	13.2	0.16 ± 0.03	0.04 ± 0.00 **^1^	0.05 ± 0.01 **	0.01 ± 0.02 **	0.06 ± 0.00 **	0.02 ± 0.02 **	0.01 ± 0.02 **	0.03 ± 0.06	0.06 ± 0.06
Farnesene	C_15_H_32_	27.3	0.73 ± 0.25	0.66 ± 0.09	0.67 ± 0.05	0.69 ± 0.12	0.89 ± 0.03	0.59 ± 0.01	0.60 ± 0.07	0.92 ± 0.35	1.34 ± 0.26
2,6,11-Trimethyldodecane	C_15_H_32_	33.1	0.75 ± 0.21	0.76 ± 0.14	0.76 ± 0.07	0.73 ± 0.24	1.06 ± 0.02	0.57 ± 0.07	0.59 ± 0.15	1.05 ± 0.33	1.66 ± 0.44
2,3,5,8-Tetramethyldecane	C_14_H_30_	34.3	0.26 ± 0.10	0.22 ± 0.04	0.23 ± 0.03	0.21 ± 0.04	0.32 ± 0.01	0.17 ± 0.02	0.19 ± 0.03	0.32 ± 0.09	0.53 ± 0.11
Palmitoleic acid	C_17_H_32_O_2_	38.5	0.59 ± 0.04	0.71 ± 0.09	0.69 ± 0.03	0.76 ± 0.06*	0.86 ± 0.06 *	0.52 ± 0.02	0.55 ± 0.07	0.63 ± 0.02	0.82 ± 0.07 *
1-propylundecylmethoxyacetate	C_17_H_34_O_3_	38.7	0.21 ± 0.01	0.25 ± 0.03	0.24 ± 0.01	0.25 ± 0.02 *	0.29 ± 0.01 **	0.17 ± 0.01 *	0.19 ± 0.01	0.20 ± 0.01	0.18 ± 0.16
Palmitic acid	C_17_H_34_O_2_	39.5	4.02 ± 0.29	4.58 ± 0.47	4.28 ± 0.15	4.18 ± 0.33	4.74 ± 0.20*	3.42 ± 0.10 *	3.68 ± 0.39	4.16 ± 0.19	5.38 ± 0.61
2,6,10-Trimethyltetradecane	C_17_H_36_	41.3	0.52 ± 0.06	0.59 ± 0.09	0.61 ± 0.02	0.68 ± 0.06	0.84 ± 0.03 **	0.56 ± 0.04	0.62 ± 0.09	0.93 ± 0.13 *	1.48 ± 0.23 *
14-Methylpentadec-9-enoic acid methyl ester	C_17_H_32_O_2_	41.6	0.13 ± 0.01	0.15 ± 0.02	0.16 ± 0.01 **	0.18 ± 0.01 *	0.23 ± 0.01 ***	0.09 ± 0.08	0.11 ± 0.09	0.12 ± 0.11	0.10 ± 0.17
Ethyl hexadecanoate	C_18_H_36_O_2_	42.7	1.88 ± 0.10	2.16 ± 0.26	2.21 ± 0.05 *	2.28 ± 0.16	2.63 ± 0.15 **	1.91 ± 0.05	2.26 ± 0.25	2.61 ± 0.12 **	3.36 ± 0.39 *
Linoleic acid	C_18_H_32_O_2_	49.5	11.74 ± 0.29	12.19 ± 0.62	11.64 ± 0.29	11.11 ± 0.60	11.35 ± 0.35	9.71 ± 0.36 **	10.10 ± 1.03	10.30 ± 0.40 *	10.51 ± 0.70
Methyl linolate	C_19_H_32_O_2_	50.1	19.46 ± 0.41	19.97 ± 0.54	18.87 ± 0.45	18.18 ± 0.92	18.64 ± 0.97	16.30 ± 0.54 **	16.40 ± 1.48	16.82 ± 0.70 *	16.69 ± 1.00 *
16-methyl-Heptadecanoic acid, methyl ester	C_19_H_38_O_2_	50.4	0.41 ± 0.57	0.84 ± 0.05	0.90 ± 0.02	0.92 ± 0.03	1.05 ± 0.05	0.78 ± 0.03	0.80 ± 0.00	0.91 ± 0.03	0.38 ± 0.66
9,12-Octadecadienoic acid, ethyl ester	C_20_H_36_O_2_	56.5	5.77 ± 0.20	5.87 ± 0.58	6.22 ± 0.21	6.23 ± 0.49	6.70 ± 0.08 **	5.66 ± 0.29	6.78 ± 0.84	7.51 ± 0.22 **	8.07 ± 0.70 *
Linolenic acid	C_20_H_34_O_2_	57.4	9.33 ± 0.37	9.42 ± 0.81	9.80 ± 0.29	9.71 ± 1.18	10.23 ± 0.15 *	9.12 ± 0.62	10.50 ± 1.39	11.33 ± 0.30 **	11.06 ± 0.75
cis-11-Eicosenoic acid	C_21_H_40_O_2_	68.5	3.69 ± 0.07	3.42 ± 0.25	3.35 ± 0.13 *	3.05 ± 0.07 **	2.71 ± 0.12 **	3.28 ± 0.19	3.20 ± 0.12 *	2.91 ± 0.25 *	2.45 ± 0.34 *
cis-13-Eicosenoic acid	C_21_H_40_O_2_	71.4	1.52 ± 0.00	1.32 ± 0.15	1.41 ± 0.02 **	1.38 ± 0.10	1.19 ± 0.11 *	1.53 ± 0.11	1.73 ± 0.10	1.67 ± 0.04 *	1.29 ± 0.22
2-methyl Eicosane	C_21_H_44_	71.7	0.38 ± 0.01	0.35 ± 0.03	0.38 ± 0.01	0.38 ± 0.02	0.35 ± 0.05	0.40 ± 0.02	0.46 ± 0.03 *	0.43 ± 0.01 **	0.35 ± 0.03
Methyl 13-eicosenoate	C_21_H_40_O_2_	75.9	26.86 ± 1.87	25.57 ± 2.87	25.61 ± 1.12	26.06 ± 2.71	23.25 ± 0.87	28.06 ± 2.77	26.16 ± 3.07	22.71 ± 0.59 *	20.15 ± 2.14 *
Methyl (Z)-13-docosenoate	C_23_H_44_O_2_	77.6	9.93 ± 1.02	9.01 ± 0.84	9.83 ± 0.38	10.64 ± 0.98	9.78 ± 1.08	11.95 ± 1.08	12.96 ± 2.26	11.72 ± 0.48	10.07 ± 1.18
Methyl nervonate	C_25_H_48_O_2_	80.7	0.48 ± 0.09	0.47 ± 0.09	0.48 ± 0.03	0.51 ± 0.13	0.45 ± 0.05	0.58 ± 0.07	0.53 ± 0.16	0.42 ± 0.01	0.35 ± 0.05
Methyl lignocerate	C_25_H_50_O_2_	81.2	0.19 ± 0.06	0.24 ± 0.03	0.30 ± 0.02 *	0.41 ± 0.11	0.54 ± 0.04 **	0.31 ± 0.02*	0.30 ± 0.09	0.29 ± 0.00 *	0.48 ± 0.01 **
24,25-Dihydroxycholecalciferol	C_27_H_44_O_3_	82.1	0.20 ± 0.04	0.19 ± 0.03	0.22 ± 0.01	0.25 ± 0.05	0.25 ± 0.03	0.29 ± 0.03 *	0.30 ± 0.10	0.27 ± 0.02	0.26 ± 0.02
Total			99.18	98.97	98.89	98.79	98.40	96.01	99.03	98.26	97.04

^1^ The *t*-test showed statistically significant difference (*** *p* <0.001, ** *p* <0.01, * *p* <0.05) compared with control (0 h).

**Table 3:** TPC and ABTS assay of UVB-treated purple kohlrabi sprouts.

	0 h	Non-UV Treated				UV Treated			
		6 h	12 h	24 h	48 h	6 h	12 h	24 h	48 h
Total phenolic compounds (mg/g)	0.62 ± 0.00	0.62 ± 0.01	0.60 ± 0.01	0.65 ± 0.01	0.61 ± 0.01	0.61 ± 0.00	0.63 ± 0.00	0.66 ± 0.00	0.67 ± 0.00
ABTS (inhibition %)	46.80 ± 4.11	46.73 ± 0.40	44.72 ± 1.05	46.29 ± 0.89	47.60 ± 0.76	46.82 ± 1.75	51.94 ± 1.31	54.06 ± 1.76 *	51.31 ± 0.18

The *t*-test showed a statistically significant difference (* *p* < 0.05) compared with the control (0 h).

## Data Availability

Data reported are available in the Supplementary Materials.

## Supplementary Materials

The following supporting information can be downloaded at: https://www.mdpi.com/article/10.3390/antiox11081443/s1, Figure S1: HPLC chromatogram of anthocyanin compounds in UVB-treated purple kohlrabi sprouts corresponding to Table 1; Figure S2. GC chromatogram of fatty acids in UVB-treated purple kohlrabi sprouts corresponding to Table 2; Figure S3. GC-TOFMS chromatogram of hydrophilic compounds in UVB-treated purple kohlrabi sprouts. Peak: 1, Pyruvic acid; 2, Lactic Acid; 3, Alanine; 4, Oxalic acid; 5, Glycolic acid; 6, Valine; 7, Urea; 8, Serine-1; 9, Ethanolamine; 10, Phosphoric acid; 11, Glycerol; 12, Leucine; 13, Isoleucine; 14, Proline; 15, Glycine; 16, Succinic Acid; 17, Glyceric Acid; 18, Fumaric Acid; 19, Serine-2; 20, Threonine; 21, β-Alanine; 22, Malic acid; 23, Aspartic Acid; 24, Methionine; 25, Pyroglutamic Acid; 26, 4-Aminobutyric Acid; 27, Threonic acid; 28, Cysteine; 29, Glutamic Acid; 30, Phenylalanine; 31, Xylose-1; 32, Xylose-2; 33, Arabinose; 34, Asparagine; 35, Xylitol; 36, Ribitol (internal standard) 37, Putrescine; 38, Glutamine; 39, Shikimic acid; 40, Citric acid; 41, Quinic acid; 42, Fructose-1; 43, Fructose-2; 44, Mannose; 45, Glucose-1; 46, Glucose-2; 47, Mannitol; 48, Lysine; 49,Tyrosine; 50, Inositol; 51, Ferulic acid; 52, Tryptophan; 53, Sinapinic acid; 54, Fructose-6-phosphate; 55, Glucose-6-phosphate-1; 56, Glucose-6-phosphate-2; 57, Sucrose; Figure S4. Correlation matrix of anthocyanins, ABTS values, and total phenolics (TPC) in UVB-irradiated purple kohlrabi sprouts. Control, purple kohlrabi sprouts grown without UVB irradiation for 0 h, 6 h, 12 h, 24 h, and 48 h; test, purple kohlrabi sprouts irradiated with UVB for 6 h, 12 h, 24 h, and 48 h; Table S1. Hydrophilic compounds quantified in UVB-treated purple kohlrabi sprouts corresponding to Figure 1.
