# Supplementary material for: Metabolic Analyses and Evaluation of Antioxidant Activity in Purple Kohlrabi Sprouts after Exposed to UVB Radiation
Source: Antioxidants (Basel). 2022 Jul 25;11(8):1443. doi: 10.3390/antiox11081443 (PMC9332045; doi:10.3390/antiox11081443)
Supplement: Supplementary file 1 [file antioxidants-11-01443-s001.zip › Supplementary Materials/Supplementary Materials Figures S1-S4.pdf]

Supplementary Materials

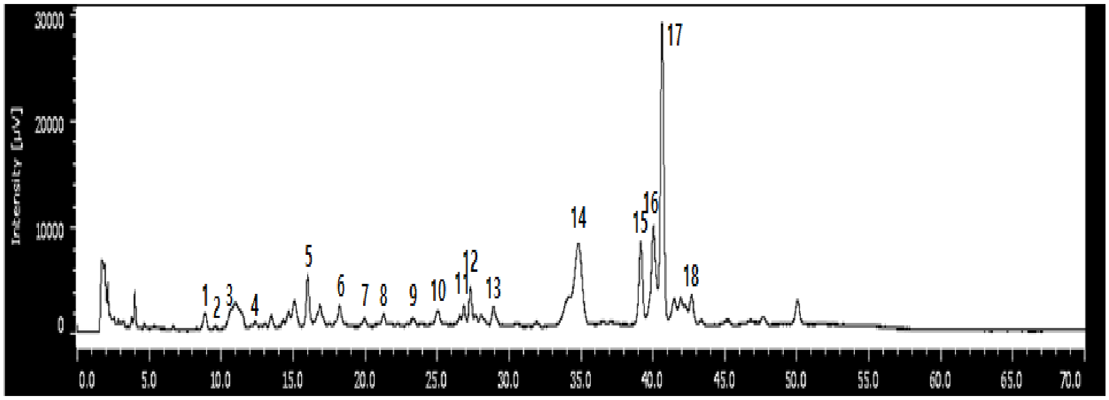

Figure S1. HPLC chromatogram of anthocyanin compounds in UVB-treated purple kohlrabi sprouts corresponding to Table 1.

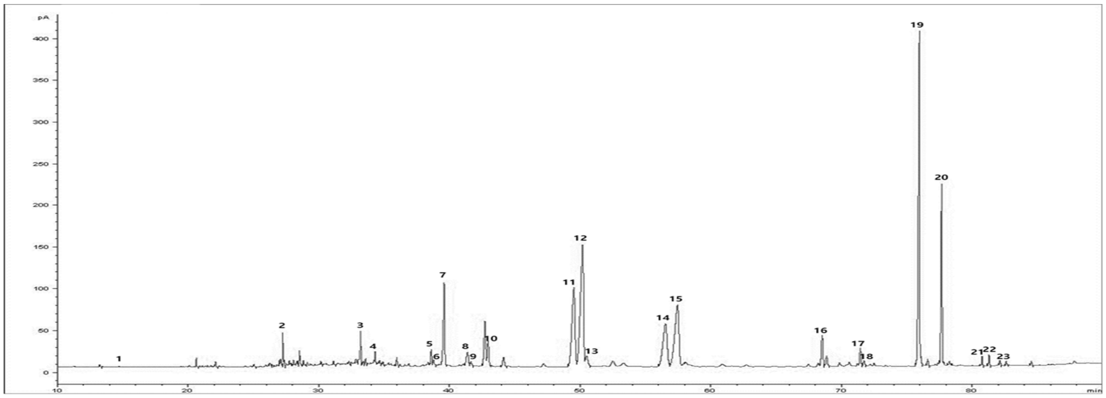

Figure S2. GC chromatogram of fatty acids in UVB-treated purple kohlrabi sprouts corresponding to Table 2.

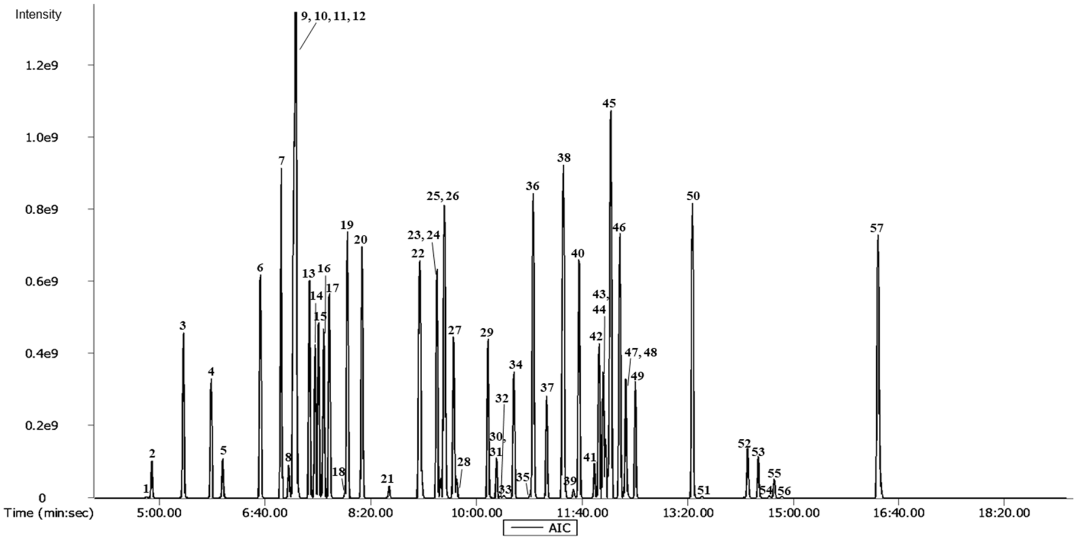

Figure S3. GC-TOFMS chromatogram of hydrophilic compounds in UVB-treated purple kohlrabi sprouts. Peak: 1, Pyruvic acid; 2, Lactic Acid; 3, Alanine; 4, Oxalic acid; 5, Glycolic acid; 6, Valine; 7, Urea; 8, Serine-1; 9, Ethanolamine; 10, Phosphoric acid; 11, Glycerol; 12, Leucine; 13, Isoleucine; 14, Proline; 15, Glycine; 16, Succinic Acid; 17, Glyceric Acid; 18, Fumaric Acid; 19, Serine-2; 20, Threonine; 21, β-Alanine; 22, Malic acid; 23, Aspartic Acid; 24, Methionine; 25, Pyroglutamic Acid; 26, 4-Aminobutyric Acid; 27, Threonic acid; 28, Cysteine; 29, Glutamic Acid; 30, Phenylalanine; 31, Xylose-1; 32, Xylose-2; 33, Arabinose; 34, Asparagine; 35, Xylitol; 36, Ribitol (internal standard) 37, Putrescine; 38, Glutamine; 39, Shikimic acid; 40, Citric acid; 41, Quinic acid; 42, Fructose-1; 43, Fructose-2; 44, Mannose; 45, Glucose-1; 46, Glucose-2; 47, Mannitol; 48, Lysine; 49,Tyrosine; 50, Inositol; 51, Ferulic acid; 52, Tryptophan; 53, Sinapinic acid; 54, Fructose-6-phosphate; 55, Glucose-6-phosphate-1; 56, Glucose-6-phosphate-2; 57, Sucrose.

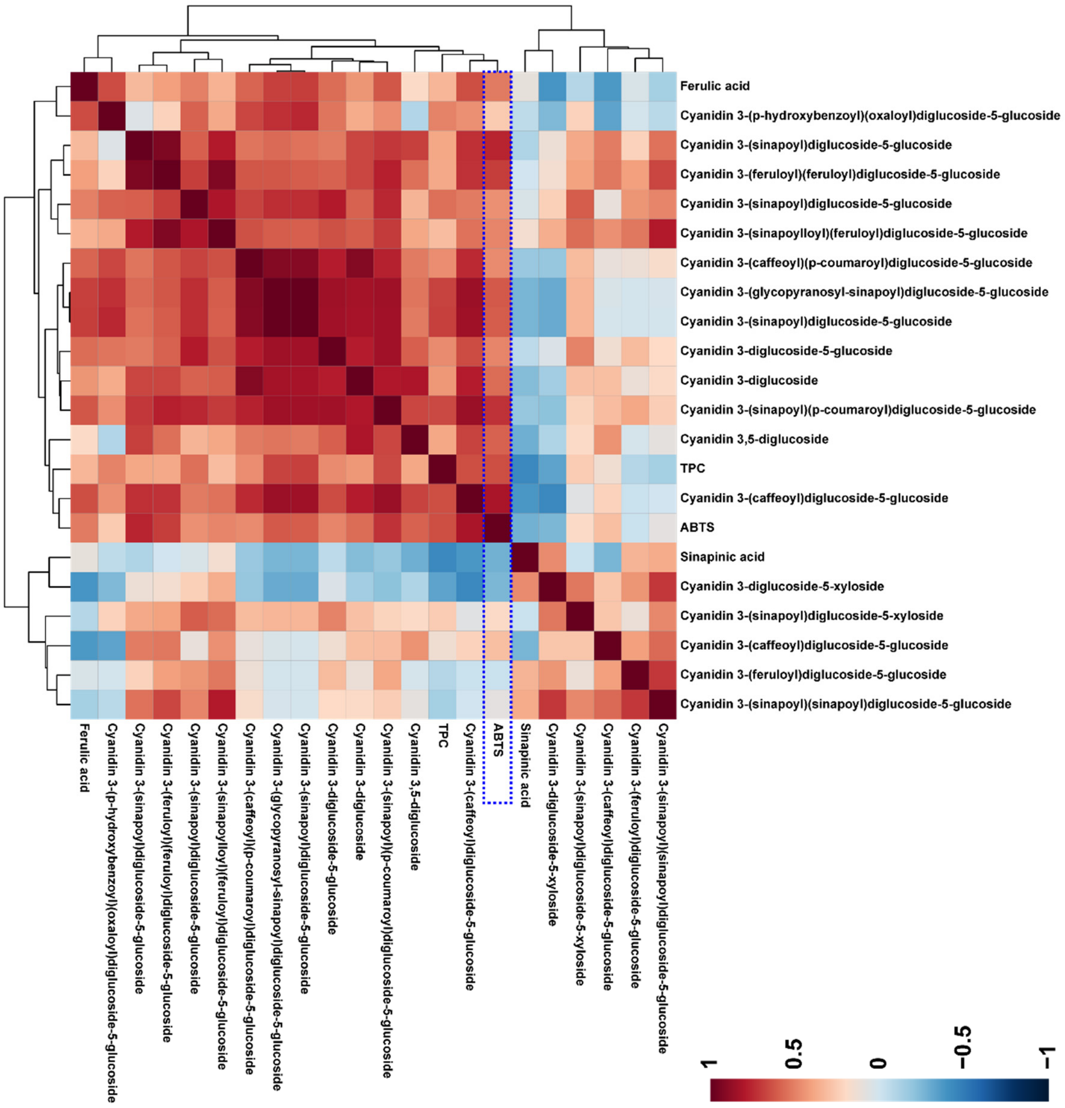

Figure S4. Correlation matrix of anthocyanins, ABTS values, and total phenolics (TPC) in UVB-irradiated purple kohlrabi sprouts. Control, purple kohlrabi sprouts grown without UVB irradiation for 0h, 6h, 12h, 24h, and 48h; test, purple kohlrabi sprouts irradiated with UVB for 6h, 12h, 24h, and 48h.
